# Susceptibility of Marmosets (*Callithrix jacchus*) to Monkeypox Virus: A Low Dose Prospective Model for Monkeypox and Smallpox Disease

**DOI:** 10.1371/journal.pone.0131742

**Published:** 2015-07-06

**Authors:** Eric M. Mucker, Jennifer Chapman, Louis M. Huzella, John W. Huggins, Joshua Shamblin, Camenzind G. Robinson, Lisa E. Hensley

**Affiliations:** 1 Virology Division, United States Army Medical Research Institute of Infectious Diseases, Fort Detrick, Maryland, United States of America; 2 Joint Pathology Center, Silver Spring, Maryland, United States of America; 3 Integrated Research Facility, National Institute of Allergy and Infectious Diseases, Fort Detrick, Maryland, United States of America; 4 Pathology Division, United States Army Medical Research Institute of Infectious Diseases, Fort Detrick, Maryland, United States of America; 5 Tulane University School of Medicine, New Orleans, Louisianna, United States of America; Emory University School of Medicine, UNITED STATES

## Abstract

Although current nonhuman primate models of monkeypox and smallpox diseases provide some insight into disease pathogenesis, they require a high titer inoculum, use an unnatural route of infection, and/or do not accurately represent the entire disease course. This is a concern when developing smallpox and/or monkeypox countermeasures or trying to understand host pathogen relationships. In our studies, we altered half of the test system by using a New World nonhuman primate host, the common marmoset. Based on dose finding studies, we found that marmosets are susceptible to monkeypox virus infection, produce a high viremia, and have pathological features consistent with smallpox and monkeypox in humans. The low dose (48 plaque forming units) required to elicit a uniformly lethal disease and the extended incubation (preclinical signs) are unique features among nonhuman primate models utilizing monkeypox virus. The uniform lethality, hemorrhagic rash, high viremia, decrease in platelets, pathology, and abbreviated acute phase are reflective of early-type hemorrhagic smallpox.

## Introduction

Variola virus (VarV) and monkeypox virus (MPXV), the etiological agent of smallpox and monkeypox diseases respectively, are members of the genus *orthopox* in the family *Poxviridae* which consists of large (about 200Kb), double stranded-DNA viruses. Through coordinated vaccination efforts, naturally occurring VarV has been eradicated. Cessation of routine vaccination has left the global population with no, or waning, immunity. Reintroduction of wild type or a modified version of variola virus into a now susceptible population would be socially and economically devastating. Moreover, there is an increased incidence of other orthopoxvirus infections such as monkeypox, cowpox, and vaccinia viruses [1]. Of these, monkeypox virus is especially worrisome because it is more frequently reported and more severe in humans [2, 3]. The outbreak in the United States in 2003 heightened our awareness and concerns as the disease was capable of infiltrating the western hemisphere [4].

Smallpox and monkeypox have a very similar clinical presentation. Both have an incubation time of approximately 10 days, followed by fever and concomitant appearance of rash. Most cases of smallpox were categorized as ordinary-type smallpox, in which a centrifugal exanthem progressed through multiple stages (e.g., macules, papules, vesicles, pustules, scabs). Mortality in ordinary smallpox was about 30%. This is in sharp contrast to the hemorrhagic form (early and late-type) of the disease, which was almost uniformly lethal and lacked progressive skin lesions. Monkeypox is not known to have a true hemorrhagic presentation in humans, although it was noted that a patient from the 2003 United States outbreak had hemorrhage within skin lesions [5]. Monkeypox is less severe than smallpox (> 10% mortality), and clinically resembles discrete ordinary or modified forms of smallpox [6]. Lymphadenopathy is thought to be characteristic of monkeypox, a feature not reported by clinicians who have previously treated smallpox afflicted individuals [6].

To date, there are no Food and Drug Administration (FDA) licensed antiviral therapies to combat a smallpox, or any other poxvirus, outbreak. Humans are the only known host of variola virus. Since exposure of humans to variola virus would be unethical and field studies with surrogates are not feasible, the development and licensure of potential countermeasures will rely on efficacy in animal models through FDA’s “Animal Rule” [7, 8]. Within these regulations, survival (decrease in mortality) is the generally accepted outcome for assessing the benefit of a biologic or therapeutic in a model system that parallels human disease [7–9]. Also, the etiologic agent at a dose and route similar to human exposure should be part of the test system. The limited access to, and restricted host range of, variola virus has precluded the development of such models. In fact, the only variola virus based model to be used for assessing efficacy of a test article is the semi-lethal intravenous macaque model [10, 11]. These issues have prompted the use of an appropriate surrogate virus, that is, one that causes smallpox-like disease in humans—namely, monkeypox virus.

Intravenous (IV) infection of macaques with MPXV is the predominant NHP model system for the development of smallpox countermeasures (reviewed by [12]). Shortly after a high dose inoculation, macaques become febrile, develop a characteristic progressive rash, viremia, and subsequently succumb to infection [10, 13, 14]. Although the model provides a good representation of severe lesional disease, it unfortunately, like variola virus in macaques, entails a large bolus of virus administered through an unnatural route. Other nonhuman primate (macaque) models utilizing MPXV, such as aerosol, intrabronchial, and intratracheal inoculation models, capture the essence of a natural infection, but still require a high dose of virus and sacrifice lesional burden for route of administration [15–18]. Moreover, all macaque models utilizing MPXV tend to have an abbreviated incubation period. Although macaques are susceptible to infection with monkeypox virus (by definition), thus far, a faithful, fulminant disease under conditions that recapitulate human infection (e.g., low dose, natural route) have not been successful in macaques.

It was the goal of our lab to either improve upon the MPXV-macaque intravenous model or to change the host to another more susceptible NHP species. We were unsuccessful in lowering the dose by administering sucrose gradient purified MPXV or a fresh preparation of MPXV that incorporated the extracellular form of the virus (Mucker, unpublished). Therefore, we turned to the common marmoset (*Callithrix jacchus*) as a potential susceptible host. Marmosets are becoming a more common host for modeling viral diseases. Such diseases include: Lassa fever, Eastern Equine Encephalitis, Ebola, Marburg, Dengue, Rift Valley Fever, Hepatitis C, and influenza [19–25]. The genetic similarity to humans, small size, availability, and relative safety are all attributes contributing to the increasing use of marmosets [26]. Outbreaks of poxvirus in marmoset colonies have been documented [27, 28]. An orthopox model utilizing calpox virus was developed concomitant with our studies and showed that marmosets were indeed susceptible to low levels of a poxvirus by a natural route [29, 30]. Calpox virus is a relatively new orthopoxvirus and was originally identified in marmosets in 2006 [28] Although resembling cowpox virus, calpox virus has yet to be adequately characterized and is not known to cause disease in humans. Monkeypox virus has been reported in captive marmosets housed in the Rotterdam Zoo in 1964 [31, 32]. Although a few marmosets were exhibiting mild signs of illness, only a single animal (one that succumbed to disease) was confirmed to have monkeypox [31, 32].

Using a dose-down strategy, we show that marmosets are highly susceptible to low doses of monkeypox virus via the intravenous route and have an incubation period (pre-clinical signs) more characteristic of monkeypox and smallpox disease.

## Materials and Methods

### Virus Preparation and Cells

The monkeypox virus strain Zaire (V79-I-005) stock was previously described [10, 14]. The virus was plaque titrated on Vero E6 cells (ATCC CRL-1586). Inoculum was diluted to the initial target dose and 1:10 dilutions of this stock were made for subsequent inoculums. All inoculums were subsequently back titrated.

### Hematology and Quantitative PCR

Hematological data was generated on an ACT 10 Beckmann Coulter using whole EDTA blood. Since “in-house” hematological reference values were unavailable, reference values from Johnson-Delaney, 1994 for white blood cells and platelets [33] and Adams et al. 2008 for lymphocyte numbers [20] were utilized. Extractions were performed using Qiagen QiAMP DNA Blood Minikit according to manufacturer’s instructions using 50uL of EDTA blood, except for the heat inactivation step (10 min @ 56°C) in lysis buffer which was extended for 1 hour to ensure inactivation of the virus Quantitative PCR was performed as previously described [10, 34].

### Plaque Reduction Neutralization Assays (PRNT) and Blood/Serum Titration

Plaque reduction neutralization assays (varying serum/plasma, constant virus) were performed on all animals pre-infection and on the lowest dosing group post infection. Briefly, serum or plasma samples were diluted 1:10 and heat inactivated for 30 minutes in a 56°C waterbath. Serially 1:4 dilutions were performed in EMEM and monkeypox virus was added for a target of 100 PFU/well. The samples were incubated at 4°C overnight.

Both the neutralization assay and blood/blood component samples were titrated in a similar manner. One hundred microliters of sample was adsorbed to decanted Vero E6 cells for one hour. A liquid overlay of EMEM containing 2% heat inactivated serum was added to each well and incubated at 37°C for 5 days. Crystal violet was used to stain and elucidate plaques.

### Nonhuman Primates and Inoculation

Eighteen adult male marmosets (*Callithrix jacchus*) weighing greater than 300 grams were screened for neutralization activity to monkeypox virus previous to infection. The inoculum was prepared as described in the “virus preparation” section and 300 uL was loaded into 1 mL syringes. Groups of three marmosets were infected intravenously via the tail vein, or saphenous vein if infection via the tail vein was unachievable. Blood from the femoral vein was acquired within two minutes of infection. Phlebotomy and physical examinations were performed every three days post exposure, except where noted. Rectal temperatures were performed on the first iteration, the latter half of the second iteration, and subsequent iterations. Microchips (BMDS) were used to identify and ascertain temperature during the first half of the second iteration, but ceased due to equipment failure. Reference temperature ranges from Johnson-Delaney, 1994 [33] were utilized and are provided.

### Necropsy

A necropsy was performed on all animals, either as soon as death occurred from infection or after humane euthanasia of terminally ill or moribund animals. All tissues were immersion-fixed in 10% neutral buffered formalin for a minimum of 21 days, according to Institute protocol.

### Histology and Immunohistochemistry

Formalin-fixed tissues for histologic examination were trimmed, processed, and embedded in paraffin according to established protocols [35]. Histology sections were cut at 5μm, mounted on glass slides, and stained with hematoxylin and eosin (H&E). Immunohistochemical staining was performed on replicate tissues sections using an Envision + kit (DAKO, Carpinteria, CA). Normal splenic tissue (USAMRIID) served as the negative control; the positive control was spleen from a known monkeypox virus infected nonhuman primate (USAMRIID); and normal rabbit serum (USAMRIID) was used as the negative control. Briefly, sections were deparaffinized in xyless, rehydrated in graded ethanol, and endogenous peroxidase activity was quenched in a 0.3% hydrogen peroxide/methanol solution for 30 min at room temperature. Slides were washed in phosphate buffered saline (PBS) then sections were incubated in the primary antibody, a non-commercial rabbit polyclonal antibody (USAMRIID) against vaccinia virus, diluted 1:3500 for 60 minutes at room temperature. Sections were washed in PBS and incubated for 30 min with Envision + rabbit secondary reagent (horseradish peroxidase-labeled polymer) at room temperature. Peroxidase activity was developed with 3,3’-diaminobenzidine (DAB), counterstained with hematoxylin, dehydrated, cleared with xyless, then coverslipped.

### Electron Microscopy

Selected sections of formalin-fixed liver were trimmed for electron microscopy, post fixed in a mixed aldehyde fixative followed by osmium tetroxide, contrasted in ethanolic uranyl acetate, dehydrated in an ascending series of ethanol, infiltrated in a mixture of propylene oxide and resin, and embedded into EMBed 812 resin. Thin sections (~80 nm) were mounted on copper EM support grids and counter stained with uranyl and lead salts. Samples were examined on a JEOL 1011 transmission electron microscope at 80kV. All supplies for electron microscopy were from Electron Microscopy Sciences (Hatfield, PA) unless otherwise noted.

### Ethics Statement

Research was conducted under in compliance with the Animal Welfare Act, PHS Policy, and other Federal statutes and regulations relating to animals and experiments involving animals. The facility where this research was conducted is accredited by the Association for Assessment and Accreditation of Laboratory Animal Care, International and adheres to principles stated in the Guide for the Care and Use of Laboratory Animals, National Research Council, 2011. All animal experiments were approved by USAMRIID’s Insitutional Animal Care and Use Committee.

Animals were housed in individual metal cages meeting current standards for the duration of the housing period in biocontainment level 3. Room environment is centrally controlled by an HVAC system that maintains room humidity and temperature. Animals were provided pelleted commercially available feed and potable water was provided at libitum from an automatic watering system. In addition, animals received supplemental foods, treats and fruits daily. Animals are provided manipulanda (toys, metal mirrors), foraging devices, treats and fruits as enrichment. Treats and extra fruits were increased while in biocontainment. Euthanasia was performed when the animal(s) met the criteria for euthanasia using a score sheet for intervention or when found moribund. The scoring systems was based on recumbency, prostration, dyspnea, and responsiveness. Animals were oberserved and scored at least twice daily by trained personnel. In addition,husbandry and general assessments were conducted at least once daily by Veterinary Division caretakers. Animals requiring euthanasia were anesthetized and subsequently euthanized with a pentobarbital based solution following the AVMA Guidelines on Euthanasia. Of the experimentally exposed animals, eleven met the criteria and six succumbed to poxvirus-related disease. The six animals that succumbed exhibited signs of disease, but did not meet criteria for euthanasia. Even with multiple checks per day, it was not possible to implement early endpoint euthanasia for all animals on this study. In addition, one animal succumbed to an experimentally unrelated condition. Anesthesia was also provided prior to performing phlebotomy. Analgesics were withheld to avoid any known and/or potential alterations of the disease process The scientific justification was approved for withholding analgesia by the IACUC, as required by applicable laws and regulation.

## Results

### Disease Development

**Fig 1:**
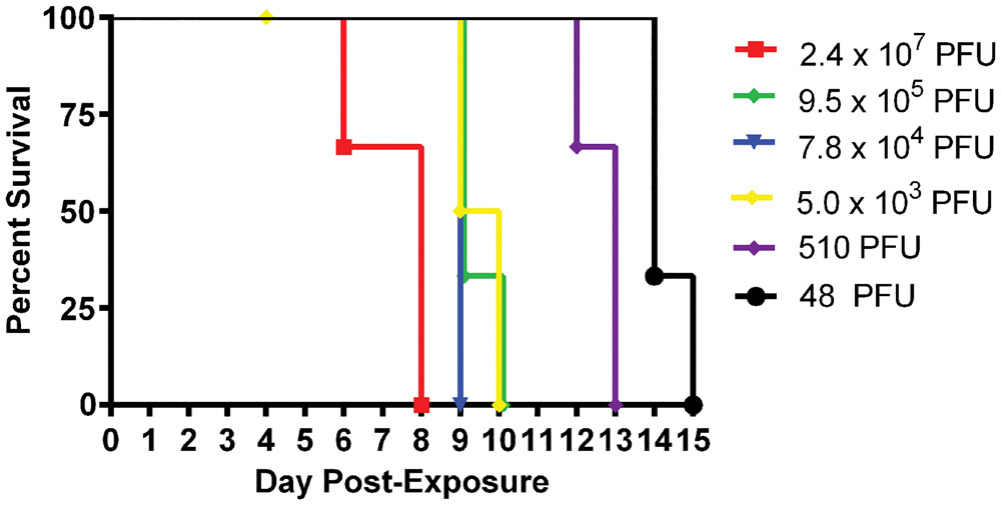
Survival curve for marmosets intravenously exposed to decreasing doses of monkeypox virus. Groups of animals (n = 3) were exposed to 10 fold reductions in viral titer in six different experiments. In order to proceed to the next lower dose, 100% of the animals had to succumb. Due to the severity of the disease, one dose (5x10^6^ PFU) was not evaluated.

**Fig 2:**
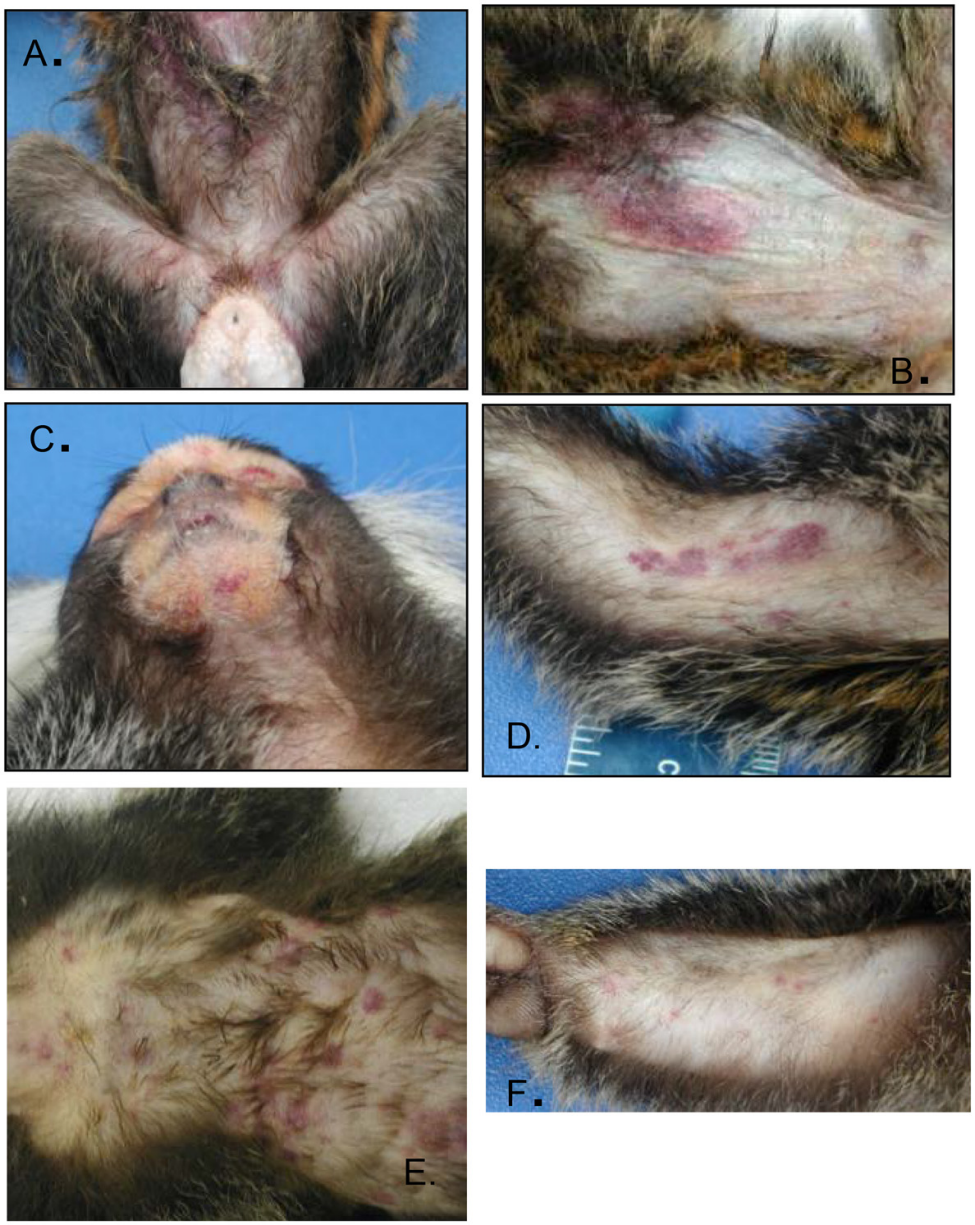
Rash presentation of marmosets intravenously exposed to decreasing doses of monkeypox virus. Notice the rash becomes more focal in nature as the dose is decreased: 2.4 x 10^7^ PFU, A, 9.5 x 10^5^ PFU, B, 7.8 x 10^4^ PFU, C, 5.0 x 10^3^ PFU, D, 510 PFU, E, and 48 PFU.

**Fig 3:**
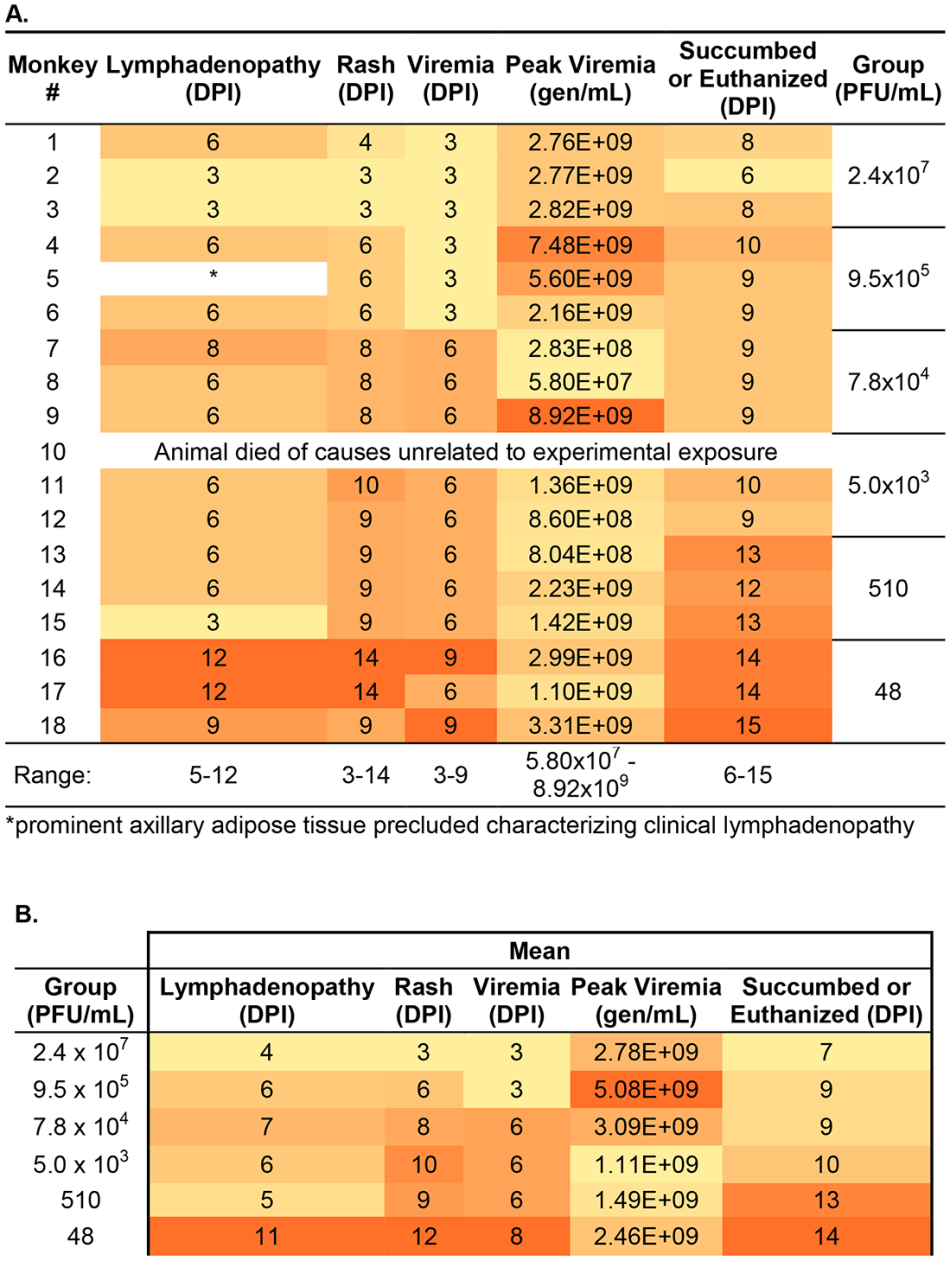
Summary of key features of disease. Summary of the key features of disease by individual NHP, A, or by group, B,. Color has been added to highlight trends, as darker shading represents an increase in magnitude for each column. DPI, day post infection.

Groups of three marmosets (a total of eighteen) were intravenously exposed to six decreasing doses of MPXV. We chose a starting target dose of 5x10^7^ PFU as this dose is commonly used in the intravenous macaque model [12]. Animals were challenged with either 2.4x10^7^, 9.5x10^5^, 7.8 x10^4^, 5.0x10^3^, 510, or 48 PFU, as established by titration of the inoculums. All animals developed a similar disease course and died or were euthanized by day 15 post-infection (Fig 1). One animal died from causes unrelated to experimental infection, and data pertaining to this animal was excluded. The major differences between the doses were the temporal onset of disease and the phenotypic presentation of rash (Figs 2 and 3). These differences can be generally categorized into three groups: (1) animals succumbing relatively quickly, characterized by generalized hemorrhagic manifestations, (2) animals that survived longer and had more focal/discrete epidermal lesions, and (3) animals that were intermediate (between 1 and 2) for both survival and rash presentation. More specifically, animals in the highest dose group had definable clinical signs on day two, decreased activity, anterior abdominal matting of haircoat, and an unkempt appearance. By day three, a cutaneous rash (generalized anterior erythema and few petechiae), significant lymphadenopathy, and pronounced lethargy were observed in two of three animals. These clinical signs became more severe, with animals becoming somnolent before succumbing to the disease by day 9. In fact, the disease was so severe that we opted to skip a challenge dose. In contrast, lymphadenopathy and rash in the lowest dose group were not observed until at least day 9 and the earliest time point for euthanasia in this group was on day 14. The lesions in this group were much more discrete and were composed of flat, well-defined lesions that appeared dark red but never progressed through the typical stages of classic orthopoxvirus disease (Fig 2). The disease manifested in animals infected with 9.5x10^5^ PFU and 510 PFU, resembled their higher or lower dose counterparts, respectively. Animals in the remaining two dosing groups (7.8 x10^4^ and 5.0x10^3^ PFU) had slightly mixed presentations, with generalized hemorrhagic manifestations and few focal lesions. The appearance of the rash and lymphadenopathy relative to the day they succumbed/euthanized tended to be more animal specific, rather than dose specific. For instance, there was variation from 0–10 days between onset of rash/lymphadenopathy and death (Fig 3).

### Viral Load and Hematology

**Fig 4:**
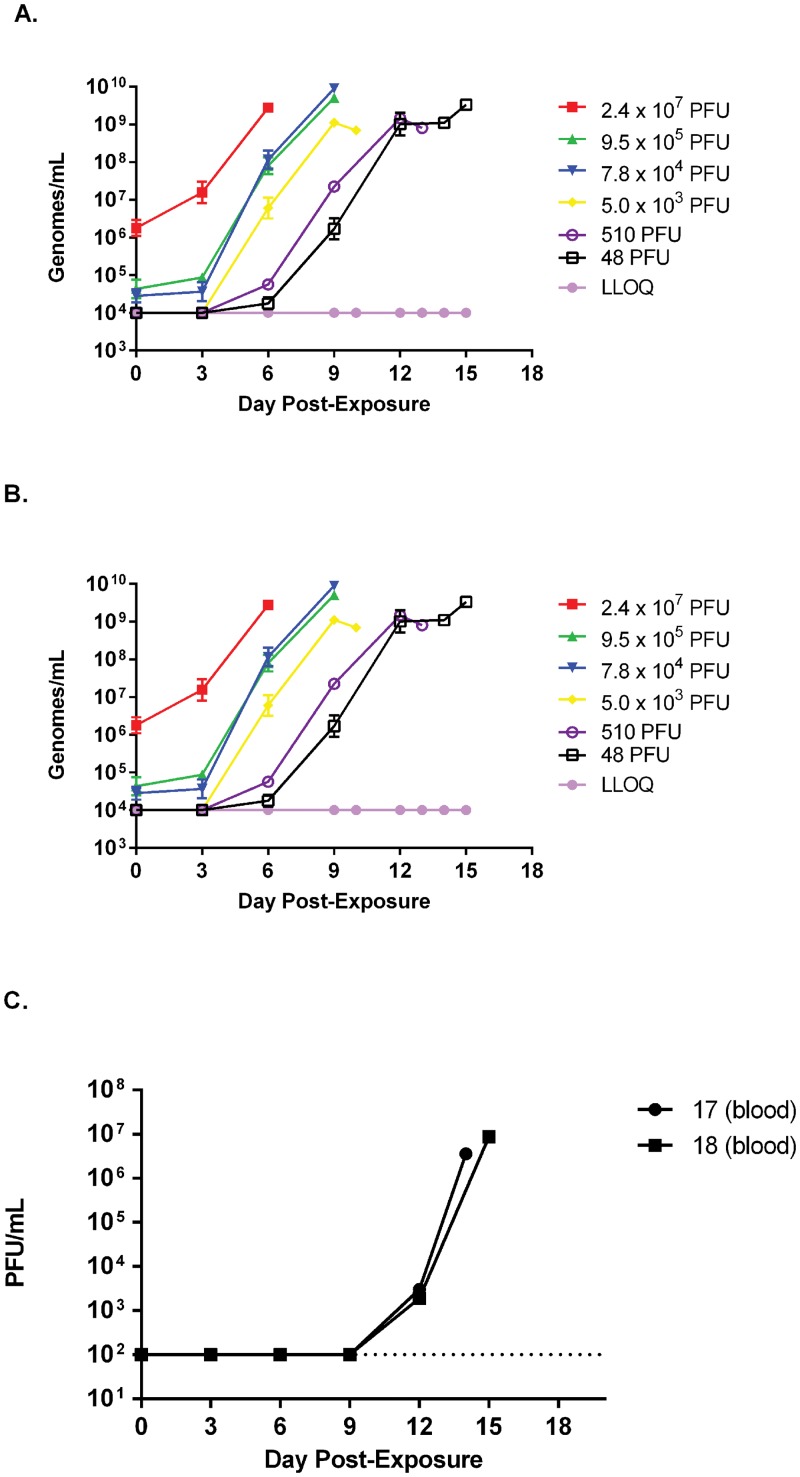
Viremia in marmosets intravenously exposed to monkeypox virus. QPCR was performed on extracted DNA from EDTA whole blood and presented as genomes per milliliter of blood, either by group, A, or individual marmosets, B. The lower limit of quantitation is also presented as a dotted line. Notice the temporal shift (dose response) in the onset of viremia. To determine if infectious virus could be detected, plaque titrations were performed from EDTA whole blood in two 48 PFU dosed animals, C.

To test whether marmosets exhibit a systemic and circulating infection, a feature of smallpox disease and reported correlate of infection in other nonhuman primate models [10, 14], we tested blood for the presence of viral genomic material using an established QPCR assay [34]. Animals were bled on days -3, 0 (post infection), 3, 6, 9, 12, 15 post infection on which QPCR and hematology was performed. Quantifiable detection of circulating viral genomes was dose dependent and ranged in individual animals from days 3 to 9 and between groups (mean) from days 3 to 8 (Figs 3, 4A and 4B). Animals in all groups produced a striking increase in circulating viral genomes (Fig 4B). This change ranged from about 3 to 5 logs, with the greatest increase generated by the lowest two challenged groups where no genomes were quantifiable until at least day 6 in one of three animals (Fig 4). The post infection bleeds (Day 0), which were acquired less than 2 minutes after inoculation, suggest some variation in dosing during inoculation (Fig 4B). Affirmation of circulating infectious virus was obtained by plaque titration of both serum and whole EDTA blood from the lowest dosing group (Fig 4C).

**Fig 5:**
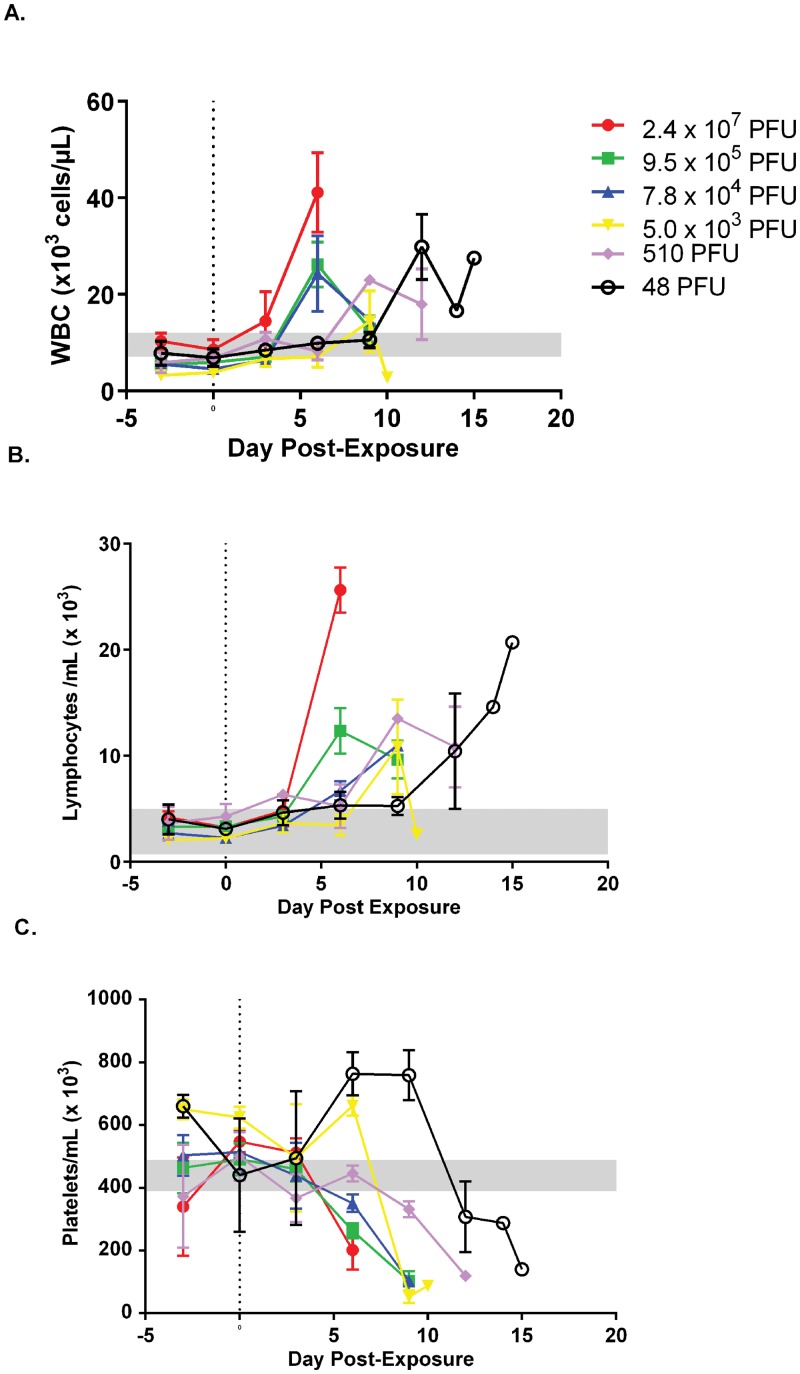
Temporal evaluation of white blood cells (WBC) and platelets in marmosets exposed to decreasing doses of monkeypox virus. Values were obtained using EDTA whole blood using a Beckman ACT 10 Hematology Analyzer. Absolute values by group are presented for WBC, A, and lymphocytes, B, and platelets, C. Notice the increase and temporal shift for the lower dose groups in reference to the total WBC and lymphocytes, A and B. Decreasing platelet numbers were also apparent. Reference ranges are from [33] (WBC and platelets) and [20] (lymphocyte #).

Mobilization of white blood cells is a hallmark of most viral infections. Like genomic viremia, rash, and lymphadenopathy, changes in the white blood cell counts were temporal, depending on dose (Fig 5A). The greatest increases occurred on samples obtained on days 6 and 9 for most animals. In the lowest dose group, all three animals had their largest increase on day 12 (Fig 5A). Changes in lymphocyte number followed the same trend, increasing over time (Fig 5B).

In most animals tested (16 of 17 animals) there was a marked decrease in the number of platelets in the last 3 to 6 days of the disease course (Fig 5C). These changes appear to be dose dependent: Animals in the highest three dose groups (8 of 9 animals) dropped below the reference range [33] between days 3 and 6 post exposure, with 7 of 8 of these animals dropping on day 6. Whereas 4 of 5 animals in the 5x10^3^ PFU and 510 PFU groups and 2 of 3 animals in the 48 PFU group crossed this threshold on day 9 and 12 post exposure, respectively. Animal #17 (48 PFU) had low values on day 0 (85,000 platelets/μL) and day 3 (69,000 platelets/μL) post exposure. Values for this animal rebounded (692,000 platelets/μL) to slightly above baseline levels on day 6 and, subsequently, dropped below the reference value on day 14. It is difficult to say whether this fluctuation was a legitimate finding in this animal or if the two early samples were falsely lowered (e.g., due to pseudo-thrombocytopenia and platelet clumping).

### Weight and Temperature

**Fig 6:**
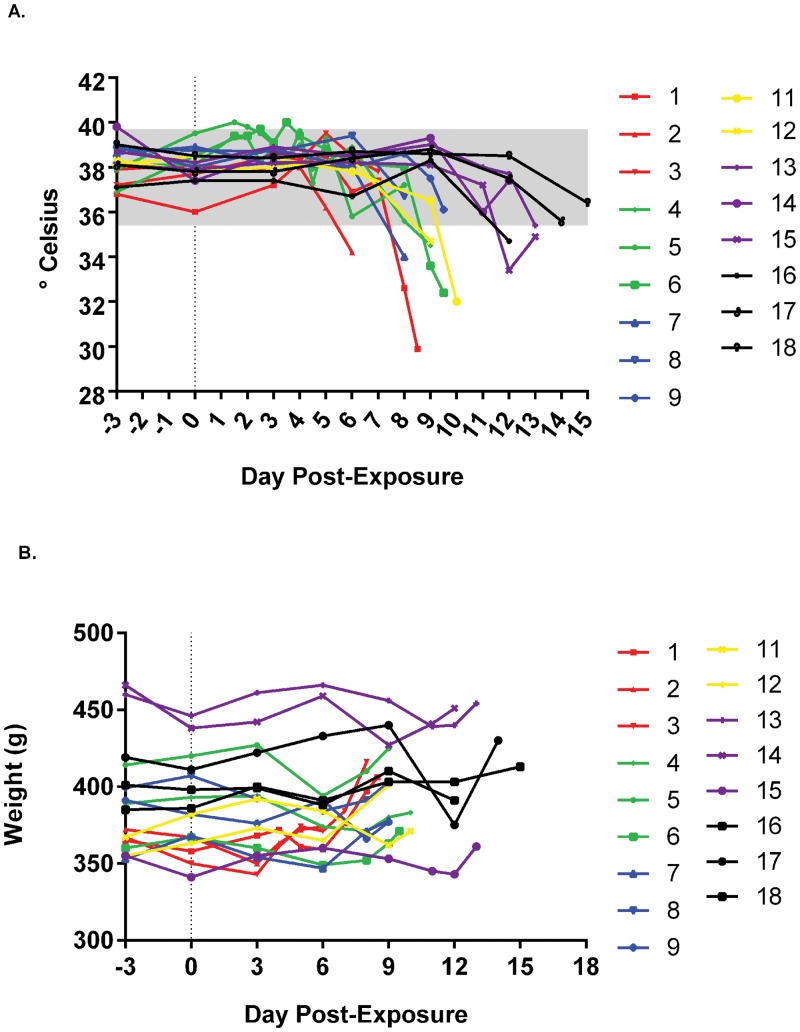
Evaluation of weights and temperature in marmosets exposed to decreasing doses of monkeypox virus. Absolute values of temperatures, A, and weight, B, are provided. Temperatures were rectally acquired except for 9.5 x 10^5^ PFU dosing group, in which subcutaneous implants (BMDS) were used until failure of the equipment (day 6 post exposure). Data for groups/individuals have been color coded to match previous graphs and normal ranges from [33], A, are provided as a shaded box.

Smallpox is known to cause a fever immediately preceding the onset of lesions. To assess whether marmosets responded in a similar manner, rectal temperatures were captured when hands-on procedures were conducted (ie., under anesthesia). After the first iteration (highest dose group), we attempted to collect more temperature data (with and without anesthesia) by implanting microchips (animals #4, #5 and #6). Unfortunately, equipment failure precluded this activity. Temperature data had a similar trend in all dosing groups. Although no discernable fever was detected (with the possible exception of animal #2 and animal #4 on days 2 and 4, respectively), which is probably due to the basal variability in marmosets and the infrequency of data capture, animals became hypothermic towards the final stages of disease (Fig 6A). On the other hand, weight tended to decrease slightly and rebound, approaching or surpassing the pre-infection weight (Fig 6B). This is most likely due to an inability of the diseased animal to adequately maintain fluid homeostasis.

### Plaque Reduction Neutralization

**Fig 7:**
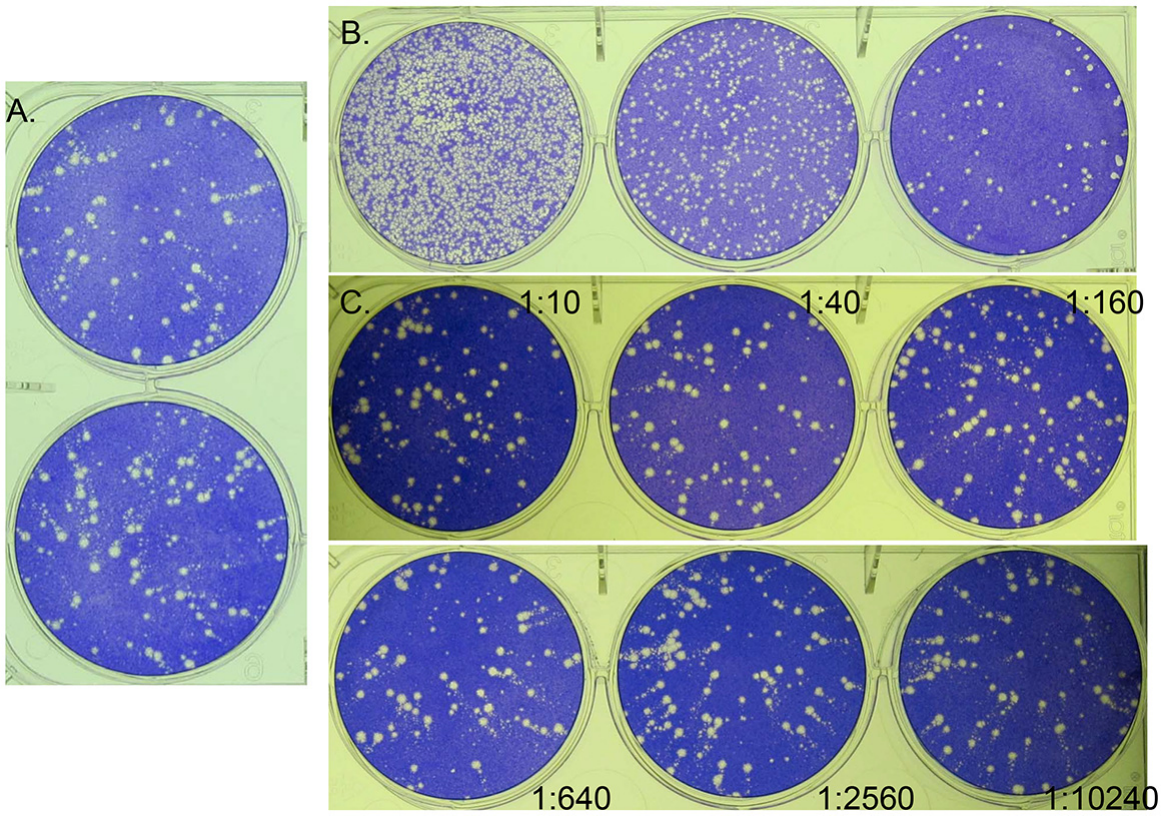
Qualitative reduction of secondary plaque formation (comets) in serum samples derived from marmosets exposed to monkeypox virus. Virus control, A; Day 14 post exposure plaque titration assay (serum), B. Neutralization assay of animal #18, 48 PFU group (heat inactivated serum), C. Notice the focal nature of the plaques from the day 14 post infection serum, B, compared to the virus control, A, indicating a potential anti-EEV effect. The cause of this effect is heat stable and exhibits a dose/dilution response; notice the increase in comets as the heat inactivated serum concentration decreases (dilution annotated by each well), C.

Samples from the low dose group were assessed for their ability to neutralize monkeypox virus on post infection days 9, 12, and terminal days (days 14 or 15). In terms of plaque counts, plasma or serum from these samples did not neutralize monkeypox virus to an appreciable degree (data not shown). It was noted that plasma/serum collected on days 9 and 12 seemed to reduce the presentation of comets (secondary plaque formation) relative to the concentration of heat inactivated serum present (Fig 7C). Comets are formed by the release of the extracellular form of poxviruses (extracellular enveloped virions, or EEV) and can be inhibited by antibody specific for the extracellular form, with or without complement ([36–40]). Samples of the same animals on Days 14 or 15 were similar to controls and showed no apparent reduction in comet formation (data not shown).

Plaque titration of the Day 14 and Day 15 serum alone produced a more focused plaque morphology, relative to the virus control (Fig 7A and 7B), suggesting an anti-EEV response. Unlike samples utilized for the neutralization assay, titration samples were not heated to inactivate complement (as this inactivates the virus). In this case, any role of complement in reducing comet formation can not be ruled out, as the heat inactivated samples did not reduce comets for Day 14 and 15 samples. It is possible that antibody neutralization (from the Day 14 and 15 samples) is complement dependent or that, because of the high titer of virus present in these samples, the antibody was not in excess and could not inhibit comets when additional virus was added for the neutralization assay. Further evaluation is needed to confirm the presence of EEV specific antibody in these samples.

### Gross Pathology

Gross pathologic findings for all animals were similar, regardless of dose group, with the exception of cutaneous lesions. All 18 animals had an erythematous rash (Fig 2) in the anterior chest, abdominal and inguinal regions, but there were varying degrees of petechiae and ecchymoses on the face, chin, chest, abdomen, axillary and inguinal areas, forearms, and scrotum, depending on dose group. There were discrete areas of hemorrhage (petechiae and ecchymoses) rather than a generalized erythematous rash in animals that survived longer (i.e. lower dose groups).

**Fig 8:**
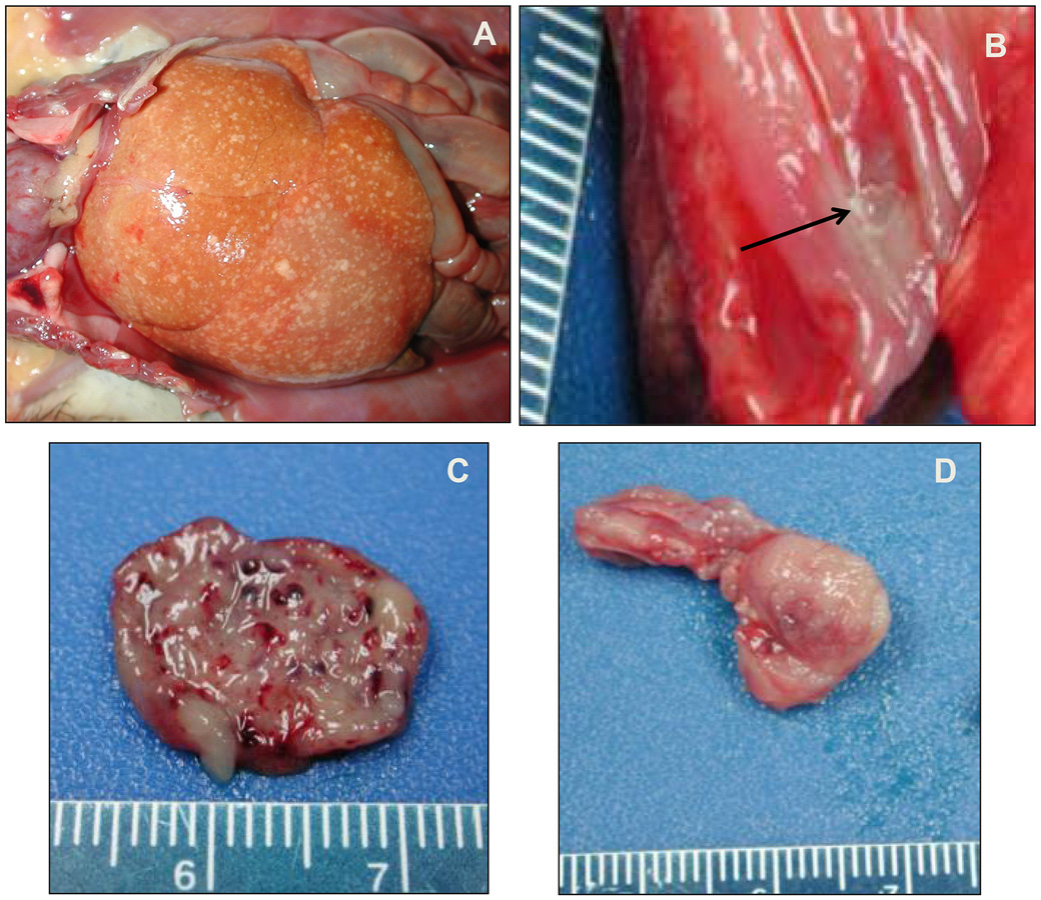
Gross pathological findings for monkeypox virus exposed marmosets. A. Liver. Animal #14 (510 PFU group). The liver is enlarged and pale with diffuse, variably sized flat, tan lesions. B. Esophagus. Animal 12 (7.8 x 10^4^ PFU group). Focal mucosal ulcer (arrow). C) Urinary bladder. Animal 4 (9.5 x 10^5^ PFU group). Multifocal mucosal hemorrhages. D. Testis. Animal #1 (2.4 x 10^7^ PFU group). Multifocal hemorrhage and necrosis.

All animals (18/18) exhibited one or more enlarged, dark red peripheral lymph nodes (axillary, inguinal, mandibular). Eight animals (8/18) had variable amounts of subcutaneous edema, and 2/18 had serosanguineous peritoneal effusion. All animals (18/18) had gross liver lesions (Fig 8A), varying from mild to marked enlargement and diffuse pallor, with variable numbers of flat, 2–5 mm diameter, white-tan foci throughout all lobes. One animal (#3) had ulcers on the mucocutaneous membrane of the lip and one animal (#12) had an esophageal ulcer (Fig 8B).

Nine (9/18) animals had hemorrhage and/or petechiae in the urinary bladder (Fig 8C). Hemorrhage was present in multiple additional sites including the testes (3/18, Fig 8D), adjacent to the kidney (1/18), in the stomach and colon (1/18), abdominal cavity (1/18), buccal mucosa (1/18), conjunctiva (1/18), and blood clots in the gallbladder (1/18). There were raised white pleural lesions in 6/18 animals, which were determined to be foci of mineralization on histologic examination and unrelated to experimental disease.

### Histology, Immunohistochemistry, and Ultrastructure

**Fig 9:**
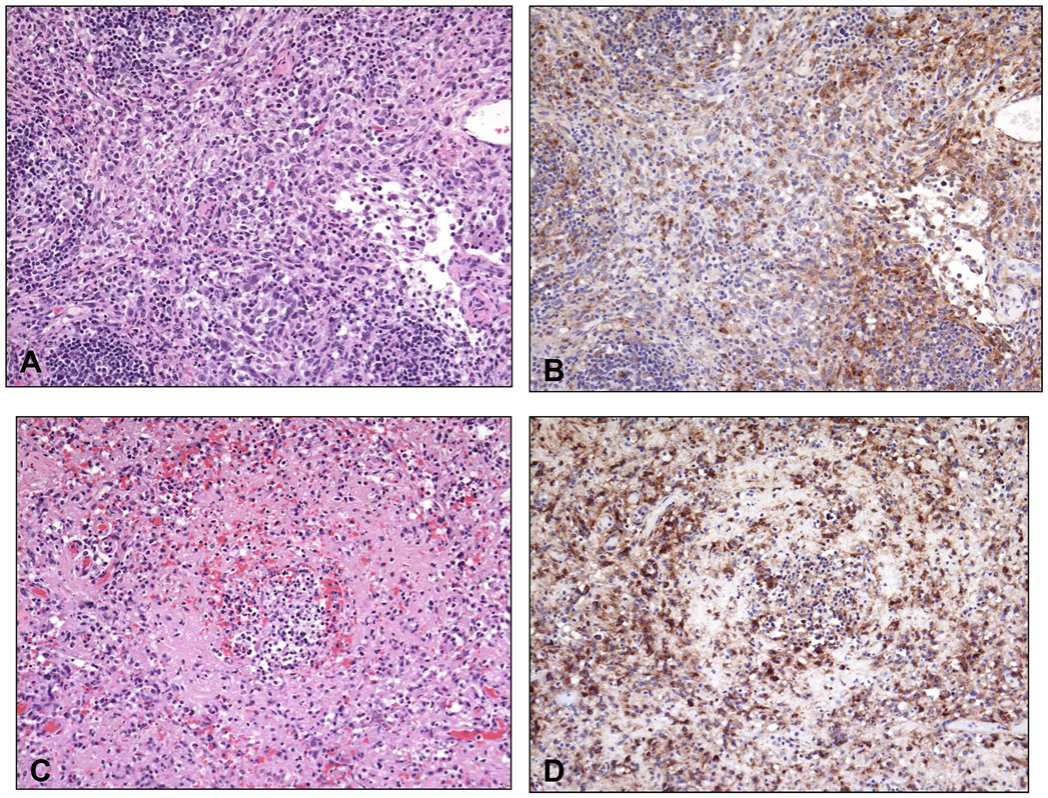
Histopathological and immunohistochemical findings in the inguinal lymph node and spleen. Histopathological and immunohistochemical findings in the inguinal lymph node (A and B, Animal #13 (510 PFU group) and spleen (C and D, Animal #16 (48 PFU group). A) There is depletion of lymphocytes and replacement by inflammatory cells (macrophages and neutrophils) admixed with necrotic cells, necrotic debris, and fibrin. B) Poxviral antigen is present predominantly in mononuclear inflammatory cells. C) Diffuse depletion of white pulp with lymphocytolysis and necrosis. D) Antigen is abundant in cellular debris and multiple cell types (mononuclear inflammatory cells, endothelial cells, supporting stromal cells). Routine HE stain (A and C). Immunoperoxidase method with hematoxylin counterstain (B and D). All at 20X.

Histopathologic lesions were not dose-dependent. All animals had lesions attributable to MPXV exposure consistently observed in the lymph nodes, spleen, liver, adrenal glands, and bone marrow (S1 Table). Lesions in the lungs and skin were also seen across all dose ranges, but varied more in severity. There was lymphoid depletion (18/18) and necrosis (14/18) within one or more lymph nodes (Fig 9A). In the spleen there was significant white pulp depletion with areas of necrosis (17/18; Fig 9C).

**Fig 10:**
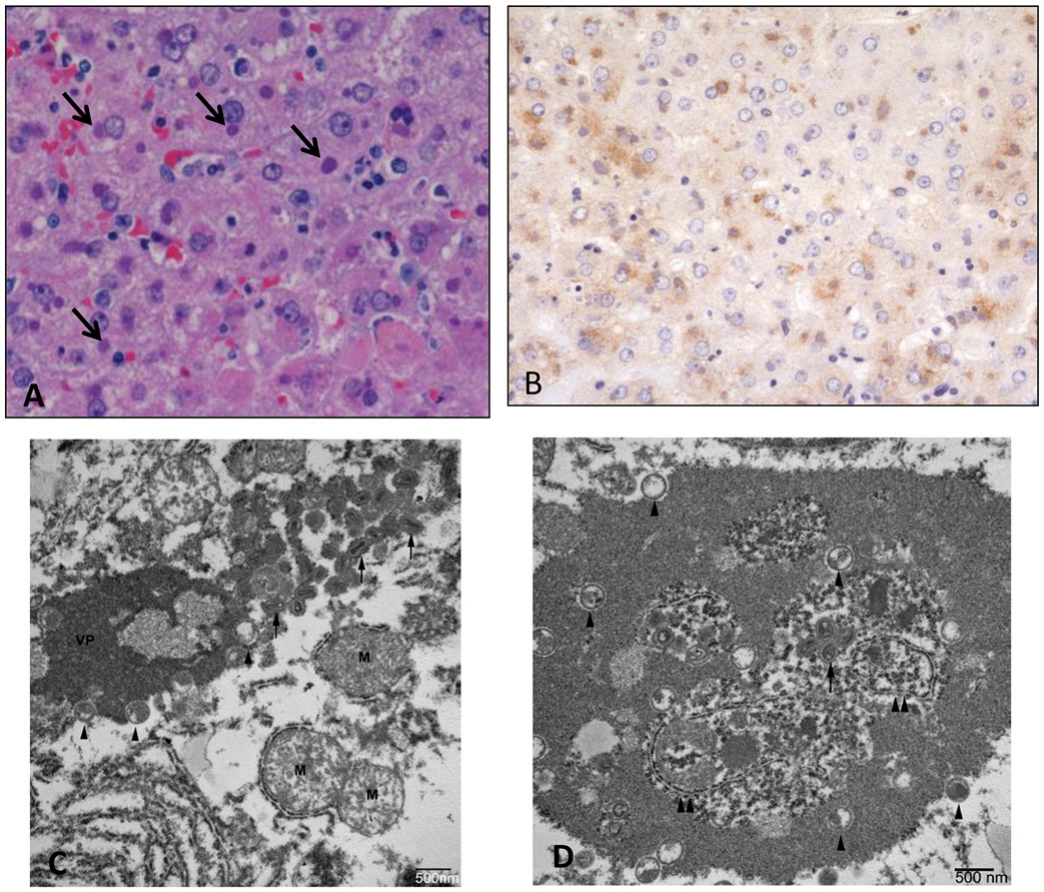
Histopathological, immunohistochemical, and electron microscopic findings in the liver. Animal #3 (2.4 x 10^7^ PFU group). A) Hepatocellular degeneration and necrosis with prominent eosinophilic intracytoplasmic inclusions (arrows). HE. B) Immunohistochemistry demonstrates vaccinia viral antigen in the liver. Immunoperoxidase method with hematoxylin counterstain. C) Transmission electron micrograph of inclusion in hepatocyte. Note the varying stages of virion from immature (arrowheads) to mature (arrows). VP—viroplasm; M—mitochondira. D) Transmission electron micrograph of inclusion in hepatocyte containing endoplasmic reticulum (double arrowheads) and free ribosomes.

**Fig 11:**
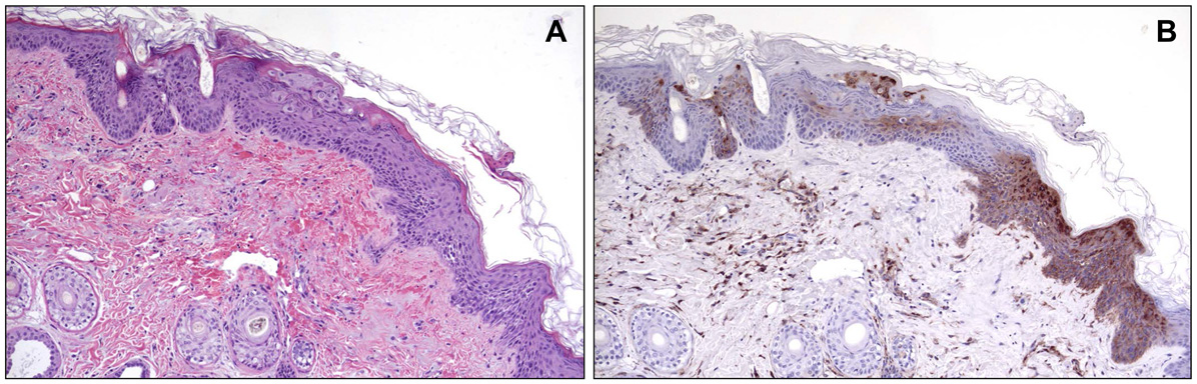
Histopathological and immunohistochemical findings in the haired skin from a lesion on the chin. Animal #9 (7.8 x 10^4^ PFU group). A) Epithelium is hyperplastic with degeneration and necrosis of epithelial cells. Syncytial cells are also present. Note hemorrhage in the underlying dermis. HE. 10X. B) Replicate section of A. Antigen is present in epithelial cells, as well as fibroblasts and inflammatory cells in the dermis. Immunoperoxidase method with hematoxylin counterstain. 10X.

**Fig 12:**
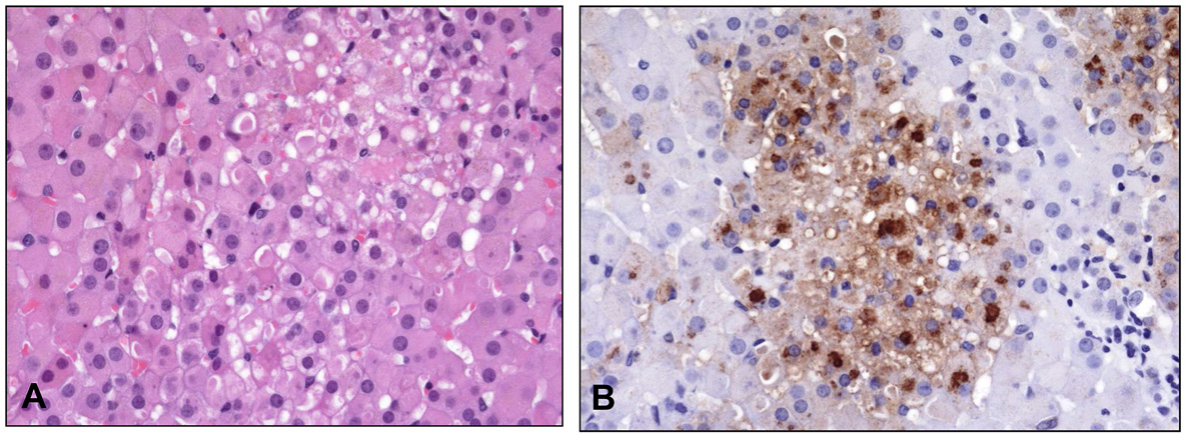
Histopathological and immunohistochemical findings in the adrenal gland. Animal #9 (7.8 x 10^4^ PFU group). A) Multifocal degeneration and necrosis of adrenal cortical cells. HE. 40X. B) Replicate section of A. Antigen is present in affected cells. Immunoperoxidase method with hematoxylin counterstain. 40X.

There was hepatocellular degeneration and loss (17/18), with most affected cells containing prominent eosinophilic intracytoplasmic inclusions (Fig 10A). There were significant bone marrow alterations with depletion of white blood cell precursors, often with areas of necrosis (17/18). The only exception, in the animal that was found dead on day 4 due to causes unrelated to experimental infection, was no significant splenic, hepatic, or bone marrow pathology likely due to the animal succumbing quickly relative to exposure. There were varying degrees of pathology in the skin and mucous membranes in all 18 animals ranging from mild epithelial hyperplasia with vacuolar degeneration and multinucleated syncytial cells, to vesicular and hemorrhagic dermatitis with necrosis (Fig 11A). In all 18 animals there were lesions in the adrenal glands, ranging from mild vacuolar degeneration to necrosis (Fig 12A) and hemorrhage. In some areas of degeneration there were prominent eosinophilic intracytoplasmic inclusions within adrenal cortical cells. Other lesions present, but less consistent across dose ranges, included hemorrhage and edema within the lungs, heart, gastrointestinal tract, genitourinary system, and meninges.

All histopathologic lesions attributed to MPXV were associated with varying amounts of immunoreactivity, with antigen identified predominantly in the cytoplasm of epithelial cells and resident/infiltrating macrophages (Figs 9B, 9D, 11B, and 12B). Hepatocytes were strongly immunoreactive with antigen concentrated in the intracytoplasmic inclusions (Fig 10B). There was immunoreactivity in tissues with no morphologic alterations as well. This was concentrated in the basal aspects of epithelial surfaces and within endothelial cells, fibroblasts, and histocytes within subepithelial and submucosal tissues and connective tissue surrounding lymph nodes and other organs.

Ultrastructural examination of hepatocytes revealed that inclusions previously noted were comprised of viroplasm and virons (Fig 10C). Virions varied in their state of maturation from membrane shells containing viroplasm to mature virions in which lateral bodies could be observed. The inclusions averaged approximately 5 x 3 microns in size though this was highly variable. Lamellar structures were occasionally seen within the inclusions, as were cellular organelles including endoplasmic reticulum and free ribosomes (Fig 10D). At least one inclusion incorporated material consistent with that described by Zaucha [41] and which is thought to be used to generate viral “shells”.

## Discussion

Here we provide evidence of the susceptibility of marmosets to monkeypox virus and propose its utility as a low dose model of smallpox/monkeypox disease. Eighteen animals were intravenously inoculated with descending doses of monkeypox virus Zaire, and all but one succumbed to a hemorrhagic-like poxviral disease by day 15. One animal died on day 4 due to causes unrelated to a poxvirus insult. Onset of clinical disease varied in a dose-dependent manner, with a delay in onset of clinical signs in the lowest dosed (48 PFU) animals until day 9 post infection. Theoretically, lower doses of monkeypox virus could have been tested. The ability to accurately quantify infectious virus in the inocula, coupled with the probability of animals not receiving any virus (being such a low dose), and the likelihood of being able to reproduce a similar outcome, are just a few reasons lower doses were not evaluated. This is the first report of a nonhuman primate model utilizing monkeypox virus with such a prolonged incubation—an incubation period similar to individuals afflicted with smallpox. Therefore, this model could help alleviate two of the major critiques concerning other test systems: 1). the high dose required to achieve a satisfactory outcome and, 2). the rapid onset of disease relative to exposure. Since the intravenous exposure eclipses the primary viremia and prodromal phase of the disease, the onset of clinical signs is generally much more rapid in other nonhuman primate models of orthopoxviral disease.

The severity of disease, based on lesion presentation, was dose dependent as well. Generalized erythema present in animals at higher doses became more focal/discrete hemorrhages at lower doses, and one animal (#18) developed short-lived macules/papules. Shortly after the onset of the manifestation of rash (0–6 days), the animals succumbed to disease. This lesion phenotype following the prolonged incubation period, in conjunction with the ascribed thrombocytopenia and gross pathology, is reminiscent of what has been reported in early-type hemorrhagic cases of human smallpox [42]. The high mortality of monkeypox in marmosets also supports this supposition, as only hemorrhagic cases of human smallpox were comparably lethal.

The appearance of circulating genomic material (viremia) prior to onset of clinical manifestations also reflects what has been demonstrated in smallpox reports for early-type hemorrhagic smallpox (for review, see [43]). Relative to the macaque, nonhuman primate models utilizing either monkeypox or variola viruses and the same PCR assay, marmosets have a greater genomic viremia with approximately a 1–2 logs more genomes detected in intravenously infected macaques [10, 14, 44, 45]; and up to two logs in those exposed by respiratory route [17].

Kramski (2010) intravenously inoculated marmosets with calpox, an isolate from a poxvirus outbreak in a colony of marmosets in Germany, with similar dosing to this report (1.25x10^7^ and 1x10^4^ PFU) that proved fatal (Kramski, et al. 2010). In contrast to our study, which clinically produced mainly hemorrhagic type disease at these doses, Kramski, et al. 2010 reported defined papular lesions (2-3mm) and Matz-Rensing et al. 2012 reported papulovesicular lesions, depending on survival time [29, 30]. In our study, animals developed petechiae and ecchymoses, and no vesicles at the comparable lower dose (approximate 10^4^ PFU), suggesting that marmosets develop a more severe disease and are likely more susceptible to monkeypox virus than calpox virus. It is possible that more lesions characteristic of ordinary type smallpox would be observed if an even lower dose of MPXV were used (e.g., <10 PFU).

Other clinical manifestations, or lack thereof, sharply contrast those reported by Kramski [29] and Matz-Rensing [30]. Animals exhibited signs of illness, such as greasy and matted haircoat, lymphadenopathy and decreased activity, in some instances six days (as little as two) before succumbing to disease. Kramski et al. 2010 did not observe clinical signs until one or two days prior to death. Another novel observation from our study is that weights in some animals increased prior to death. The increase is more noticeable in the highest challenge group where subcutaneous edema was present in 2/3 animals. This phenomenon would suggest abrogated absorption and fluid imbalance as the cause of weight gain prior to death.

Monkeypox virus induced a similar high genomic load as those induced by intravenous calpox virus (Kramski, et al. 2010). In contrast, Kramski et al 2010 reported a range of 10^6^ to 10^9^ genome equivalents per milliliter, whereas in our study 4 of 6 marmosets had greater than 10^9^ genomes per milliliter, with the lowest and highest levels being 5.8x10^7^ and 8.9x10^9^ genomes per milliliter, respectively. It is possible that these differences are due to assay-to-assay variation and/or that the virus had more time (6–10 versus 4–7 days) in which to replicate. It is important to note that blood could not be obtained for all animals at the time of death and that the maximum genome load reported for these animals may be an underestimate. Finally, high blood genome levels coincide with an increase in white blood cells. This suggests that a majority of the virus is most likely cell associated.

The susceptibility of marmosets to both calpox virus and monkeypox virus implies a favorable virus–host interaction. It is tempting to assume that marmosets are immunologically deficient relative to all poxviruses, but there is no empirical evidence to support this notion. On the contrary, evidence actually supports that outcome is more likely poxvirus specific. The outbreak reported by Gough, et al. 1982 [27] is a good case in point. In this report a Tanapox-like virus infected multiple New World nonhuman primates, including marmosets. All the animals cleared the poxvirus insult, but 6 animals succumbed to a secondary bacterial infection. But in the case where marmosets are experimentally exposed to MPXV or calpox virus, death is almost certain. The difference in virulence between poxviruses in a given host is not surprising as poxviruses have adapted to certain host(s), such as the restriction of variola and camelpox viruses to humans and camelids respectively, and this is clearly illustrated by the susceptibility of macaques to MPXV relative to VARV. Although the question of the marmoset as a favorable host for variola virus modeling can only be solved empirically, the aforementioned relationship becomes an advantage when contemplating the use of marmosets for variola virus infection, i.e., a less favorable virus host relationship between variola virus and marmosets (less virulent relative to monkeypox virus in marmosets) could allow more immune control and have more clinical attributes of ordinary smallpox.

Classic poxviral lesions (enanthema and exanthema) were not features of MPXV disease in marmosets in the current study, in contrast to calpox in marmosets where papulovesicular lesions were described [30]. Cutaneous lesions in marmosets in our study were characterized by petechiae, ecchymoses, and hemorrhage, similar to lesions described in the hemorrhagic form of smallpox in humans [42]. Lymphoid depletion and necrosis, and hematopoietic necrosis were also observed in our study, and are features reported in hemorrhagic smallpox in macaques and humans [46]. In contrast, lymphoid hyperplasia was reported in the marmoset calpox model, suggesting an immune response in the calpox model but ineffective immune response in the marmoset MPXV model. Supporting this notion, blood samples from animals that survived the longest (lowest dose group) failed to effectively neutralize monkeypox virus. It was noted that the samples qualitatively exhibited an effect on virus spread (comets). Further studies are required to elucidate the true neutralizing effect, as the assays were performed in the absence of complement, utilized only IMV, and were without consideration for inherent viral antigen (virus antigen present from the disease). Hepatocellular degeneration with single cell necrosis and intracytoplasmic inclusion bodies were features common to both the marmoset model of calpox and of MPXV.

Much like the VARV macaque model, there was widespread immunohistochemical staining of macrophages in various tissues of the MPXV marmoset model, suggesting that this cell type is fundamental in the pathogenesis of the disease. Other antigen positive tissues common to both the hemorrhagic variola macaque model and the MPXV marmoset models include epithelial, testicular, adrenocortical, and hepatic, as well as endothelial cell immunopositivity. One key difference, however, is that secondary bacterial infection is thought to potentiate hemorrhagic smallpox in macaques [46] but secondary bacterial infection was not observed in our study with marmosets. Our findings reflect those found by Bras, 1952 in humans, in which he found that in a majority of the 177 fatal smallpox cases, pathology revealed an absence of bacteria [42]. It should be noted that these patients received antibiotics and yet there were still cases of hemorrhagic disease.

Since there are no active cases of smallpox and because of ethical considerations, new medical countermeasures must be tested and approved utilizing animal models via what is commonly referred to as the “Animal Rule” [7, 8]. The Food and Drug Administration has released draft guidelines to help meet requirements for therapeutic licensure [9]. Among the recommendations within this document are the utilization of the etiologic agent at a dose reflective of human disease, producing an appropriate disease course, in a susceptible host. Nonhuman primate models utilizing variola virus or monkeypox virus have yet to meet these criteria. In this report, we provide evidence for the susceptibility of marmosets to monkeypox virus with a prolonged incubation period more indicative of smallpox and utilizing one of the agents (monkeypox virus) to which therapeutics are being developed, increasing the confidence that the therapeutic will be efficacious in a real world scenario. In essence, the monkeypox marmoset model complements the intravenous macaque model because it adds “low dose” and “extended incubation” to the test system. Therefore, the MPXV/marmoset model has the potential to bring the scientific community one step closer to fulfilling the “Animal Rule”.

As the MPXV/marmoset model is in its infancy of development, further studies are required to optimize and produce a pragmatic model. These experiments include defining an optimized route of inoculation (respiratory route), characterizing the pathogenesis and host responses, and assessing the predictive nature of the model. Additionally, model development with marmosets should include trials with variola virus. The importance of low dose variola model cannot be understated. This feat has yet to be accomplished and could accelerate the approval of prospective therapeutics.

## Supporting Information

S1 Table Summary of pathological features of marmosets exposed to monkeypox virus. (XLSX)

## References

[1] Shchelkunov SN. An increasing danger of zoonotic orthopoxvirus infections. PLoS pathogens. 2013;9(12):e1003756 10.1371/journal.ppat.1003756 24339772 PMC3855571

[2] Reynolds MG, Damon IK. Outbreaks of human monkeypox after cessation of smallpox vaccination. Trends in microbiology. 2012;20(2):80–7. 10.1016/j.tim.2011.12.001 . 22239910

[3] Rimoin AW, Mulembakani PM, Johnston SC, Lloyd Smith JO, Kisalu NK, Kinkela TL, et al Major increase in human monkeypox incidence 30 years after smallpox vaccination campaigns cease in the Democratic Republic of Congo. Proceedings of the National Academy of Sciences of the United States of America. 2010;107(37):16262–7. Epub 2010/09/02. 10.1073/pnas.1005769107 20805472 PMC2941342

[4] Reed KD, Melski JW, Graham MB, Regnery RL, Sotir MJ, Wegner MV, et al The detection of monkeypox in humans in the Western Hemisphere. The New England journal of medicine. 2004;350(4):342–50. Epub 2004/01/23. 10.1056/NEJMoa032299 . 14736926

[5] Sejvar JJ, Chowdary Y, Schomogyi M, Stevens J, Patel J, Karem K, et al Human monkeypox infection: a family cluster in the midwestern United States. The Journal of infectious diseases. 2004;190(10):1833–40. Epub 2004/10/23. 10.1086/425039 . 15499541

[6] Jezek Z, Szczeniowski M, Paluku KM, Mutombo M. Human monkeypox: clinical features of 282 patients. The Journal of infectious diseases. 1987;156(2):293–8. Epub 1987/08/01. . 3036967 10.1093/infdis/156.2.293

[7] FDA. Approval of New Drugs When Human Efficacy Studies Are Not Ethical or Feasible 2013.

[8] FDA. Approval of Biological Products When Human Efficacy Studies Are Not Ethical or Feasible 2013.

[9] FDA. Draft Guidance for Industry Animal Models—Essential Elements to Address Efficacy Under the Animal Rule. 2009.

[10] Huggins J, Goff A, Hensley L, Mucker E, Shamblin J, Wlazlowski C, et al Nonhuman primates are protected from smallpox virus or monkeypox virus challenges by the antiviral drug ST-246. Antimicrobial agents and chemotherapy. 2009;53(6):2620–5. Epub 2009/04/08. 10.1128/AAC.00021-09 19349521 PMC2687232

[11] Mucker EM, Goff AJ, Shamblin JD, Grosenbach DW, Damon IK, Mehal JM, et al Efficacy of tecovirimat (ST-246) in nonhuman primates infected with variola virus (Smallpox). Antimicrobial agents and chemotherapy. 2013;57(12):6246–53. 10.1128/AAC.00977-13 24100494 PMC3837858

[12] Parker S, Buller RM. A review of experimental and natural infections of animals with monkeypox virus between 1958 and 2012. Future virology. 2013;8(2):129–57. 10.2217/fvl.12.130 23626656 PMC3635111

[13] Hooper JW, Thompson E, Wilhelmsen C, Zimmerman M, Ichou MA, Steffen SE, et al Smallpox DNA vaccine protects nonhuman primates against lethal monkeypox. Journal of virology. 2004;78(9):4433–43. Epub 2004/04/14. 15078924 10.1128/JVI.78.9.4433-4443.2004 PMC387704

[14] Jordan R, Goff A, Frimm A, Corrado ML, Hensley LE, Byrd CM, et al ST-246 antiviral efficacy in a nonhuman primate monkeypox model: determination of the minimal effective dose and human dose justification. Antimicrobial agents and chemotherapy. 2009;53(5):1817–22. Epub 2009/02/19. 10.1128/AAC.01596-08 19223621 PMC2681496

[15] Goff AJ, Chapman J, Foster C, Wlazlowski C, Shamblin J, Lin K, et al A novel respiratory model of infection with monkeypox virus in cynomolgus macaques. Journal of virology. 2011;85(10):4898–909. Epub 2011/03/11. 10.1128/JVI.02525-10 21389129 PMC3126178

[16] Johnson RF, Dyall J, Ragland DR, Huzella L, Byrum R, Jett C, et al Comparative analysis of monkeypox virus infection of cynomolgus macaques by the intravenous or intrabronchial inoculation route. Journal of virology. 2011;85(5):2112–25. Epub 2010/12/15. 10.1128/JVI.01931-10 21147922 PMC3067809

[17] Nalca A, Livingston VA, Garza NL, Zumbrun EE, Frick OM, Chapman JL, et al Experimental infection of cynomolgus macaques (Macaca fascicularis) with aerosolized monkeypox virus. PloS one. 2010;5(9). Epub 2010/09/24. 10.1371/journal.pone.0012880 20862223 PMC2942837

[18] Stittelaar KJ, van Amerongen G, Kondova I, Kuiken T, van Lavieren RF, Pistoor FH, et al Modified vaccinia virus Ankara protects macaques against respiratory challenge with monkeypox virus. Journal of virology. 2005;79(12):7845–51. Epub 2005/05/28. 10.1128/JVI.79.12.7845-7851.2005 15919938 PMC1143678

[19] Carrion R Jr., Brasky K, Mansfield K, Johnson C, Gonzales M, Ticer A, et al Lassa virus infection in experimentally infected marmosets: liver pathology and immunophenotypic alterations in target tissues. Journal of virology. 2007;81(12):6482–90. Epub 2007/04/06. 10.1128/JVI.02876-06 17409137 PMC1900113

[20] Adams AP, Aronson JF, Tardif SD, Patterson JL, Brasky KM, Geiger R, et al Common marmosets (Callithrix jacchus) as a nonhuman primate model to assess the virulence of eastern equine encephalitis virus strains. Journal of virology. 2008;82(18):9035–42. 10.1128/JVI.00674-08 18614636 PMC2546911

[21] Carrion R Jr., Ro Y, Hoosien K, Ticer A, Brasky K, de la Garza M, et al A small nonhuman primate model for filovirus-induced disease. Virology. 2011;420(2):117–24. Epub 2011/10/01. 10.1016/j.virol.2011.08.022 21959017 PMC3195836

[22] Omatsu T, Moi ML, Hirayama T, Takasaki T, Nakamura S, Tajima S, et al Common marmoset (Callithrix jacchus) as a primate model of dengue virus infection: development of high levels of viraemia and demonstration of protective immunity. The Journal of general virology. 2011;92(Pt 10):2272–80. 10.1099/vir.0.031229-0 . 21697346

[23] Smith DR, Bird BH, Lewis B, Johnston SC, McCarthy S, Keeney A, et al Development of a novel nonhuman primate model for Rift Valley fever. Journal of virology. 2012;86(4):2109–20. 10.1128/JVI.06190-11 22156530 PMC3302397

[24] Bright H, Carroll AR, Watts PA, Fenton RJ. Development of a GB virus B marmoset model and its validation with a novel series of hepatitis C virus NS3 protease inhibitors. Journal of virology. 2004;78(4):2062–71. Epub 2004/01/30. 14747571 10.1128/JVI.78.4.2062-2071.2004 PMC369465

[25] Moncla LH, Ross TM, Dinis JM, Weinfurter JT, Mortimer TD, Schultz-Darken N, et al A novel nonhuman primate model for influenza transmission. PloS one. 2013;8(11):e78750 10.1371/journal.pone.0078750 24244352 PMC3828296

[26] Mansfield K. Marmoset models commonly used in biomedical research. Comparative medicine. 2003;53(4):383–92. . 14524414

[27] Gough AW, Barsoum NJ, Gracon SI, Mitchell L, Sturgess JM. Poxvirus infection in a colony of common marmosets (Callithrix jacchus). Laboratory animal science. 1982;32(1):87–90. . 6281571

[28] Matz-Rensing K, Ellerbrok H, Ehlers B, Pauli G, Floto A, Alex M, et al Fatal poxvirus outbreak in a colony of New World monkeys. Veterinary pathology. 2006;43(2):212–8. 10.1354/vp.43-2-212 . 16537943

[29] Kramski M, Matz-Rensing K, Stahl-Hennig C, Kaup FJ, Nitsche A, Pauli G, et al A novel highly reproducible and lethal nonhuman primate model for orthopox virus infection. PloS one. 2010;5(4):e10412 10.1371/journal.pone.0010412 20454688 PMC2861679

[30] Matz-Rensing K, Stahl-Hennig C, Kramski M, Pauli G, Ellerbrok H, Kaup FJ. The pathology of experimental poxvirus infection in common marmosets (Callithrix jacchus): further characterization of a new primate model for orthopoxvirus infections. Journal of comparative pathology. 2012;146(2–3):230–42. 10.1016/j.jcpa.2011.06.003 . 21783202

[31] Gispen R, Verlinde JD, Zwart P. Histopathological and virological studies on monkeypox. Archiv fur die gesamte Virusforschung. 1967;21(2):205–16. . 4298424 10.1007/BF01241445

[32] Peters JC. An epizootic of monkeypox at Rotterdam Zoo. International Zoo Yearbook. 1966;6(1):274–5.

[33] Johnson-Delaney CA. Primates. The Veterinary clinics of North America Small animal practice. 1994;24(1):121–56. Epub 1994/01/01. . 8109071 10.1016/s0195-5616(94)50007-x

[34] Kulesh DA, Baker RO, Loveless BM, Norwood D, Zwiers SH, Mucker E, et al Smallpox and pan-orthopox virus detection by real-time 3'-minor groove binder TaqMan assays on the roche LightCycler and the Cepheid smart Cycler platforms. Journal of clinical microbiology. 2004;42(2):601–9. Epub 2004/02/10. 14766823 10.1128/JCM.42.2.601-609.2004 PMC344443

[35] Prophet EB MB, Arrington JB, Sobin LH. Laboratory Methods for Histotechnology. Washington, D.C.: Armed Forces Institute of Pathology; 1992.

[36] Vanderplasschen A, Mathew E, Hollinshead M, Sim RB, Smith GL. Extracellular enveloped vaccinia virus is resistant to complement because of incorporation of host complement control proteins into its envelope. Proceedings of the National Academy of Sciences of the United States of America. 1998;95(13):7544–9. Epub 1998/06/24. 9636186 10.1073/pnas.95.13.7544 PMC22678

[37] Benhnia MR, McCausland MM, Moyron J, Laudenslager J, Granger S, Rickert S, et al Vaccinia virus extracellular enveloped virion neutralization in vitro and protection in vivo depend on complement. Journal of virology. 2009;83(3):1201–15. Epub 2008/11/21. 10.1128/JVI.01797-08 19019965 PMC2620895

[38] Lustig S, Fogg C, Whitbeck JC, Moss B. Synergistic neutralizing activities of antibodies to outer membrane proteins of the two infectious forms of vaccinia virus in the presence of complement. Virology. 2004;328(1):30–5. Epub 2004/09/24. 10.1016/j.virol.2004.07.024 . 15380355

[39] Chen Z, Earl P, Americo J, Damon I, Smith SK, Zhou YH, et al Chimpanzee/human mAbs to vaccinia virus B5 protein neutralize vaccinia and smallpox viruses and protect mice against vaccinia virus. Proceedings of the National Academy of Sciences of the United States of America. 2006;103(6):1882–7. 10.1073/pnas.0510598103 16436502 PMC1413659

[40] Chen Z, Earl P, Americo J, Damon I, Smith SK, Yu F, et al Characterization of chimpanzee/human monoclonal antibodies to vaccinia virus A33 glycoprotein and its variola virus homolog in vitro and in a vaccinia virus mouse protection model. Journal of virology. 2007;81(17):8989–95. Epub 2007/06/22. 10.1128/JVI.00906-07 17581986 PMC1951440

[41] Zaucha GM, Jahrling PB, Geisbert TW, Swearengen JR, Hensley L. The pathology of experimental aerosolized monkeypox virus infection in cynomolgus monkeys (Macaca fascicularis). Laboratory investigation; a journal of technical methods and pathology. 2001;81(12):1581–600. Epub 2001/12/14. . 11742030 10.1038/labinvest.3780373 PMC9827346

[42] Councilman WT, Magrath GB, Brinckerhoff WR. The Pathological Anatomy and Histology of Variola. The Journal of medical research. 1904;11(1):12–135. Epub 1904/02/01. 19971593 PMC2104636

[43] Fenner F. Smallpox and its eradication. Geneva: World Health Organization; 1988 xvi, 1460 p. p.

[44] Earl PL, Americo JL, Wyatt LS, Eller LA, Whitbeck JC, Cohen GH, et al Immunogenicity of a highly attenuated MVA smallpox vaccine and protection against monkeypox. Nature. 2004;428(6979):182–5. Epub 2004/03/12. 10.1038/nature02331 . 15014500

[45] Earl PL, Americo JL, Wyatt LS, Espenshade O, Bassler J, Gong K, et al Rapid protection in a monkeypox model by a single injection of a replication-deficient vaccinia virus. Proceedings of the National Academy of Sciences of the United States of America. 2008;105(31):10889–94. Epub 2008/08/06. 10.1073/pnas.0804985105 18678911 PMC2495015

[46] Wahl-Jensen V, Cann JA, Rubins KH, Huggins JW, Fisher RW, Johnson AJ, et al Progression of pathogenic events in cynomolgus macaques infected with variola virus. PloS one. 2011;6(10):e24832 Epub 2011/10/15. 10.1371/journal.pone.0024832 21998632 PMC3188545

